# Riders of a Modern-Day Ark

**DOI:** 10.1371/journal.pbio.0060024

**Published:** 2008-01-29

**Authors:** Virginia Gewin

## Abstract

Faced with catastrophic declines of amphibian species around the globe, scientists have launched the Amphibian Ark to ensure the global survival of critically endangered species with programs designed to fight their most immediate threat, the deadly chytrid fungus.

Amphibians may not seem the hardiest of creatures, but they have roamed Earth for 360 million years—a span including at least two major Ice Ages and four warming, interglacial periods. Yet their ability to evolve in concert with an ever-changing environment may not be enough to survive a world now dominated by human activity. Over 1,800 amphibian species, one-third of all known species, are threatened with extinction, according to the Global Amphibian Assessment [1]. Countless other, yet-undescribed, species may never have their place on Earth documented. Of all amphibians—toads, salamanders, newts, and caecilians—the frog's prevalence renders it at greatest risk.

Habitat destruction is, without a doubt, the top chronic cause of amphibian declines. Introduced exotic species, commercial trade, UV-B radiation, pesticides, and global warming are also well-documented threats. But something much more ominous—the emergence of a deadly fungus that causes sudden widespread population crashes—has compelled the conservation community to take drastic measures.

“The loss of much of an entire vertebrate class is unlike anything we've seen since the extinction of the dinosaurs,” says Kevin Zippel (see Figure 1), one of dozens of scientists backing a rescue that borders on the biblical—with a name to match. The Amphibian Ark (http://www.amphibianark.org), a plan to create a collective of hundreds of rescue facilities hosted primarily at zoos and aquariums around the world, will house and captively breed roughly 500 amphibian species deemed most at risk of extinction from the fungus. The Ark, with its focus on captive breeding, is the highest-profile initiative of the US$400 million Amphibian Conservation Action Plan (ACAP), devised two years ago by amphibian specialists desperate to save these species from the emerging fungal threat [2]. Over half of ACAP's long-term budget, however, is dedicated to identifying and safeguarding critical regions with high biodiversity value and their freshwater resources [3].

**Figure 1:**
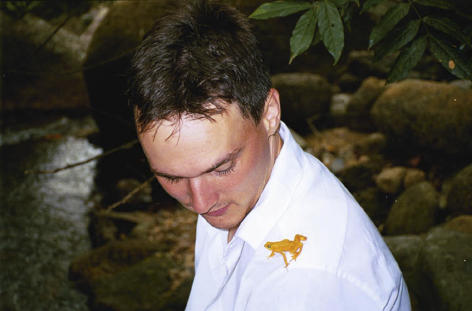
Ark Program Director Kevin Zippel and Friend (Photo credit: Corinne Richards)

In the near term, it is the Ark's estimated US$50 million 5-year cost that Ark sponsors—World Association of Zoos and Aquariums (WAZA), the World Conservation Union (IUCN) Conservation Breeding Specialist Group, and the IUCN Amphibian Specialist Group—hope to secure by designating 2008 the Year of the Frog and launching an educational and fund-raising campaign. Currently, North American zoos are prepared to manage just ten amphibian species, while the global zoo community could, at best, house 50 species. Thus, conservationists say they must act at an unprecedented international scale to protect species by preventing outbreaks of the widespread, deadly fungus.

## Controversial Conservation Challenge

“Saving amphibians is the biggest species conservation challenge in the history of humanity,” says Zippel, the Amphibian Ark's program director. Time is of the essence as species disappear—most disturbingly—from even the most pristine natural areas. Indeed, the Amphibian Ark is an unsettling symbol that the very nature of conservation is changing. Conservation efforts must now address global, not just regional, threats for which safe havens must be artificially created. And just because an area has received protection from development, for example, doesn't mean it is safe. “Protected areas don't protect you from pathogens,” says Peter Daszak, Executive Director of the Consortium for Conservation Medicine based in New York City and one of the original team to first fingerprint the fungus. And, says Joe Mendelson, herpetology curator at Zoo Atlanta in Georgia, simply conserving lands and conducting research wastes valuable time that does nothing to stave swift, fungus-fueled extinctions. “I didn't get into this business to be a paleontologist,” says Mendelson, who estimates he's already overseen the extinction of dozens of Central American species he was the first to describe.

The Ark is an expensive stop-gap measure, but some see the alternative as an unacceptable moral failure. “Ethically and morally, we have to make some attempt to save some of these species,” says Cynthia Carey, amphibian disease specialist at the University of Colorado, Boulder. Yet, even the most stalwart Ark supporters admit that captive breeding programs in general have a very low success rate and that the chances of returning species to the wild are not good, at least in the foreseeable future. And tending to the specific needs of each species brings its own challenges. The 500 most at-risk species that could benefit from captive-breeding programs right now represent 500 unique sets of breeding conditions and potential problems. For example, some tropical amphibians will only lay eggs in the water of tree-dwelling bromeliads—difficult conditions to recreate in a laboratory setting. Beyond these technical hurdles, Zippel acknowledges that the Ark's success will, at least in part, be based on the return of species to the wild. “If we only succeed with the captive component,” he says, “we will have failed.”

Over 1,800 amphibian species, one-third of all known species, are threatened with extinction.

The Ark is not without its critics. “I believe in doing in situ conservation, as opposed to putting animals in boxes and allowing them to go extinct in the wild,” says Jean-Marc Hero, Deputy Director for the Centre for Innovative Conservation Strategies at Griffith University in Queensland, Australia. As such, he contends that efforts should focus primarily on fieldwork. Ark organizers insist that in situ conservation efforts will not suffer as a result of the Ark. For example, in situ efforts are under way—most notably headed by the Arlington, Virginia–based Conservation International (CI)—to protect further losses of habitat, especially in cloud mountain forest areas that are currently chytrid free, such as Sri Lanka. CI has raised a couple of million dollars (of the hundreds of millions needed to do this fieldwork). As well, a number of smaller regional projects headed by local nonprofit, academic, and government groups aim to not only protect habitat but also to learn how to breed endemic amphibians in case the fungus arrives.

But, as of yet, the Ark is the primary ACAP priority actively raising funds from new sources. The first corporate Ark sponsors have been announced—Clorox and Prism have each donated funds to adopt an endangered amphibian species—indicating that a new level of much-needed support is gaining traction.

J. Alan Pounds, resident scientist at the Monteverde Cloud Forest Preserve in Costa Rica, is concerned that the Ark's fungus focus may overshadow the other threats and compromise a rare educational opportunity by sticking to an overly simple story. If the public views the Ark as “the solution,” he says, it could undermine the much-needed political will to raise the support and resources necessary to combat environmental deterioration also taking a toll. “This so-called ark is more like a fleet of lifeboats floating indefinitely on perilous seas,” says Pounds. Captive breeding programs have a place, he says, but much more needs to be done, including understanding how species' physical stress and resilience are related to environmental change—as well as susceptibility to the fungus.

## Deadly Disease

The fungus causing all this trouble—the so-called chytrid fungus, Batrachochytrium dendrobatidis—was first found only ten years ago in protected rainforests of Australia and Panama [4]. Thought to have originated in Africa [5], it has since been found throughout most of the world. Asia and Madagascar are the two landmasses that appear to be chytrid-free. Once the fungus invades a susceptible amphibian's permeable skin, it causes chytridiomycosis—and a rapid, grisly death. The infected animal's skin peels away, ultimately rendering it lethargic and rigid, unable to blink or flip over when touched. Amazingly, no one yet knows how the fungus spreads, or—even more remarkably—how it kills amphibians.

“We still don't know, but strongly suspect, that it probably lives on things other than amphibians,” says Ross Alford, an evolutionary ecologist at James Cook University in Queensland, Australia. Once it gets into a population of susceptible frogs, says Alford, it spreads quickly. In the late stages of disease, the population of fungus on each infected frog probably releases hundreds of millions of zoospores.

Ironically, B. dendrobatidis is a weak organism, easily killed by bacterial compounds—which allows it to be extinguished rather quickly in captive collections. However, treating individual frogs in the wild, or dumping antibacterial compounds in the wild aren't realistic solutions. And, unfortunately, the chytrid fungus has one disarming strength: the unique ability to infect a disturbingly wide range of amphibian species. Even those species that are not susceptible to the disease can at least serve as a carrier of the fungus.

B. dendrobatidis may have already stamped out species. “It may be that B. dendrobatidis is like the 1918 flu pandemic—it could be the really big one that merits this much attention,” says Mike Lannoo, biologist at Indiana University School of Medicine in Indianapolis. Other pathogens—existing or emerging—are a looming, unknown threat. “We know now there is a chytrid pandemic, but we cannot assume every disease decline is chytrid and quit looking for other pathogens,” says Alan Pessier, a pathologist at the Zoological Society of San Diego in California. “Amphibians are the last big group of animals for which we don't have good pathology information,” he adds.

The fungus causing all this trouble—the so-called chytrid fungus, Batrachochytrium dendrobatidis—was first found only ten years ago in protected rainforests of Australia and Panama.

B. dendrobatidis has hit especially hard in Central America, the Andes range of South America, western North America, and Australia. Clues about how the fungus spreads are beginning to emerge. Karen Lips, an ecologist at Southern Illinois University in Carbondale, and her colleagues can predict how the fungus spreads in a wave-like pattern, moving 28–100 km per year [6]. For the past 12 years, Wake and his students have traced the march—pond by pond—of B. dendrobatidis through Sequoia Kings Canyon National Park in central California, causing him to rethink his once-held belief that chytrid exists everywhere as an opportunist attacking already declining populations. But he says that no nonamphibian hosts, which could be harboring the fungus, have yet been found. More recently, Pounds also suggested the fungus could be triggered—possibly by global climate change—justifying the need to better understand all the factors at play.

**Figure 2:**
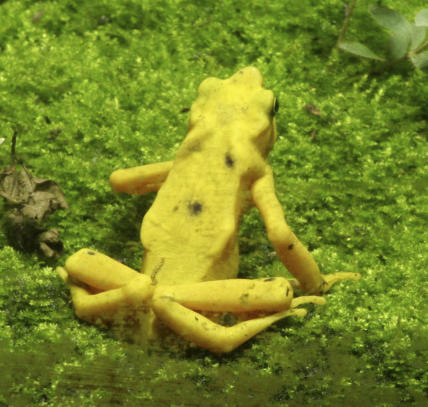
Enigmatic Amphibian Declines The Panamanian golden frog (above) is one of more than 100 species of disappearing harlequin frogs. Over 67% of the 110 tropical harlequin frog species have suffered catastrophic declines, and likely some extinctions, in the past 20 years. The B. dendrobatidis fungus's fingerprints implicated it as the sole offender [12]. Pounds' assertion that global climate change set the stage for a B. dendrobatidis outbreak sheds light on the complex inner workings of the tropical ecosystem—and offers an explanation to resolve the climate-chytrid paradox that the fungus's preferred cooler thermal range was opposite of the current climate trends. Indeed, most tropical extinctions have occurred in years that were unusually warm. While all the threats undoubtedly take a toll, for many of the visually stunning, enigmatic tropical species, the fungus can deal the final blow.

Pounds and colleagues suggested that global warming has shifted temperatures toward the growth optimum of the fungus—between 17 and 25 °C—triggering a tropical B. dendrobatidis outbreak, even though the fungus thrives in cooler temperatures [7] (Figure 2). In this model, warming increases cloud cover in many midelevation areas, making days cooler (at least at the microhabitat scale) by blocking sunlight, and making nights warmer by trapping heat. Temperatures in the lowlands are too high, while those in the highlands are too low to maintain the fungus's thermal optimum, unlike those at midelevation levels—offering an explanation for the curiously higher extinction rate in midelevation amphibian species.

Recent work in California has revealed a spatial association between pesticide use, wind patterns, and the locations of disappearing amphibian population [8]. Carlos Davidson, Director of the Environmental Studies program at San Francisco State University in California, doubts that pesticides alone are causing declines simply because they occur at very low levels in the frogs. Although he has not identified a mechanism, he suspects pesticide exposure compromises the frogs' immune system, making them susceptible to the B. dendrobatidis.

## Assembling a Modern-Day Ark

Despite the likely interactions from multiple threats, the Ark remains focused on those species most at risk from the fungus, and organizers are prioritizing species based on their biological importance to ecosystem, phylogenetic or taxonomic uniqueness, social role, educational value, and breeding knowledge. Ark organizers hope that many of the 1,200 institutions under the umbrella of WAZA will leverage their resources and claim responsibility for at least one local species. Unfortunately, the more prosperous zoos tend to be in the westernized world, which lack huge concentrations of amphibian species. But western institutions are being asked to support field projects in the more amphibian biodiverse countries.

Gordon Reid, United Kingdom–based President of the WAZA Council, says the response from member organizations has been strong, but it is too early to tally how individual institutions will contribute. “I certainly want to be ambitious. By the end of the Year of the Frog, I'd like to at least have agreements stating which institutions will commit to which species—even if we don't have the facilities in place,” says Reid.

Fortunately, it's not hugely expensive to set up biosecure facilities. Ironically, the Amphibian Ark is a loose collective of ships, or more precisely, refrigerated shipping containers, which come outfitted with temperature controls. They are easily made biosecure, and they are cheaper than new construction. Furthermore, these mini-labs are available world-wide and they're mobile—keeping costs low.

Training people in biosecure techniques and quarantine protocol, however, is an equally important task. Quarantined animals must be kept away from an established collection for at least 2–3 months at B. dendrobatidis optimum temperatures in order to best detect B. dendrobatidis, if present. A sterile swab swiped along the amphibian belly can then be sent to laboratories able to detect the fungus through genetic analysis. The tests, which cost US $25 per sample, can place a burden on smaller zoos, since tests must be repeated multiple times over a year of quarantine before new animals can join an existing zoo collection. If a frog is infected, it is separated from the group and put into baths of one of several antifungal drugs, then retested to make sure it is cleared of infection. Although it may take a couple of treatments, animals can be cured within six weeks.

Herculean efforts will be needed to ensure the long-term viability of amphibian populations. The biggest hurdle—faced by all conservation projects—is changing people's behavior so the threats no longer exist.

Pessier has been impressed with the adoption of biosecure standards at US zoos so far. “When the recommendations for quarantine first came up, I didn't expect that zoos would be receptive. Not only have zoos met the challenges of quarantine, but they've surprised me with their openness to establishing breeding programs in other countries,” he says. While there are still hold-outs, Pessier says momentum is building within the zoo community to adopt these practices.

## Rearing Resistance

Proper quarantine and biosecurity techniques are likely the only way to prevent the fungus's further spread—particularly to the chytrid-free areas. Remarkably, some species in the wild appear resistant to the deadly fungus. Amphibians may be declining in large pockets of the southeastern United States, but there are no chytrid outbreaks. In Australia, 50 species are known to be infected with B. dendrobatidis, but half of these species haven't experienced a major population effect. Pounds says the fact that at least some species may be evolving resistance to the chytrid fungus offers the best hope yet for amphibian survival. Still, the reservoir of infected, yet unaffected, amphibians in the environment remains a serious threat [9].

Given the prevalence of B. dendrobatidis and the logistical improbability of treating individual frogs, acquiring—or somehow imparting—resistance is the primary approach. Russel Poulter, researcher at the University of Otago Frog Group in Dunedin, New Zealand, claims that a well-known antibacterial compound, chloramphenicol, may not only kill the fungus but also impart resistance. Preliminary work indicates that attempts to reinfect frogs after treating the initial fungal infection never rose to significant pathology for the nine species of frogs tested so far. “What we hope that means is that we can induce an immune resistance in a frog,” he says, eager to test more species. Despite Poulter's optimism, chloramphenicol—believed to be a human carcinogen—may find little environmental application.

Reid Harris, a disease ecologist at James Mason University in Harrisonburg, Virginia, is exploring the possibility of harnessing antifungal peptides produced by bacteria already found on frog species [10,11]. Harris is cautiously optimistic that this may provide some help—even if it's a firewall-type approach. “It's way too soon to be advocating spraying bacteria out of airplanes, but if chytrid moves in a predictable wave pattern, we could possibly get in front of the wave, inoculate those frog populations in its path,” he says.

Still others are focused on genetic approaches. In Colorado, Carey has watched cured boreal toads die once returned to the wild and re-infected. “We're trying to figure out what genes we need to breed for to improve the resistance of our animals to the fungus,” she says. Directed breeding, however, will no doubt have other genetic consequences. “In selecting these genes, we know we'll lose others,” she says, “which may also be critical for long-term viability of any population.”

**Figure 3:**
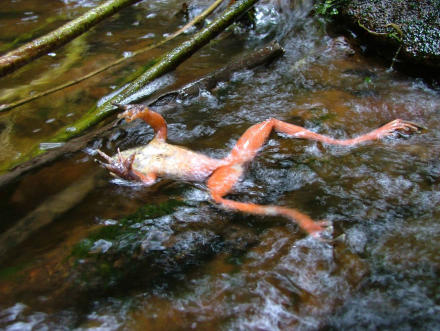
Human Impacts A chytrid-infected frog (above). Humans surely have a vested interested in keeping amphibian species alive and well—not the least of which is the potential for one day finding amphibian-based cures for human diseases. In recent years, scientists have found potential antibiotics [13] and even a potential HIV-blocking compound [14], now in clinical trials, in the thin layer of permeable frog skin. And amphibian losses will no doubt have major impacts on ecosystems. Amphibians often make up the largest biomass of any vertebrates in some ecosystems. As frogs disappear, a fundamental chink in the food chain ripples throughout the system, ultimately causing the decline of those animals that eat frogs [15]. (Photo credit: Forrest Brem)

While conservationists debate which of these, or other, drastic measures will help contain the fungus, even more Herculean efforts will be needed to ensure the long-term viability of amphibian populations. The biggest hurdle—faced by all conservation projects—is changing people's behavior so the threats no longer exist (Figure 3). The Ark itself will only affect human behavior inasmuch as it succeeds in educating the public about the urgent as well as long-term causes of decline.

Perhaps, most importantly, the amphibian crisis has put a new perspective on how conservation efforts must address ecosystem health. As climate change shifts the range of species, putting pathogens into contact with new hosts or aiding disease spread, Daszak says conservation must mature to deal with the problems. On this there is agreement. Unless human behavior changes to mitigate environmental deterioration, amphibian rescue efforts will have been for naught.
